# Carprofen alleviates Alzheimer-like phenotypes of 5XFAD transgenic mice by targeting the pathological hallmarks induced by amyloid-β aggregation

**DOI:** 10.1038/s41598-023-36167-4

**Published:** 2023-07-05

**Authors:** Donghee Lee, Ji Eun Ryoo, Seungpyo Hong, Hye Yun Kim, YoungSoo Kim

**Affiliations:** 1 Department of Pharmacy, College of Pharmacy, Yonsei University, Incheon, 21983 Republic of Korea; 2 Yonsei Institute of Pharmaceutical Sciences, College of Pharmacy, Yonsei University, Incheon, 21983 Republic of Korea; 3 Yonsei Frontier Lab, Yonsei University, Seoul, 03722 Republic of Korea; 4 Pharmaceutical Sciences Division, School of Pharmacy, University of Wisconsin-Madison, Madison, WI USA; 5 Wisconsin Center for NanoBioSystems, University of Wisconsin-Madison, Madison, WI USA; 6 Department of Integrative Biotechnology and Translational Medicine, Yonsei University, Incheon, 21983 Republic of Korea

**Keywords:** Protein folding, Protein aggregation

## Abstract

Alzheimer’s disease (AD) is characterized by misfolding, oligomerization, and accumulation of amyloid-β (Aβ) peptides in the brain. Aβ monomers transform into Aβ oligomers, which are toxic species, inducing tau hyperphosphorylation and the downstream effects on microglia and astrocytes, triggering synaptic and cognitive dysfunctions. The oligomers then deposit into Aβ plaques, primarily composed of β-stranded fibrils, required for definitive AD diagnosis. As amyloid burden plays the pivotal role in AD pathogenesis, many efforts are devoted in preventing amyloidosis as a therapeutic approach to impede the disease progression. Here, we discovered carprofen, a non-steroidal anti-inflammatory drug, accelerates Aβ aggregating into fibrils and increases Aβ plaques when intraperitoneally injected to 5XFAD transgenic mouse model. However, the drug seems to alleviate the key Alzheimer-like phenotypes induced by Aβ aggregation as we found attenuated neuroinflammation, improved post-synaptic density expression, associated with synaptic plasticity, and decreased phosphorylated tau levels. Carprofen also rescued impaired working memory as we discovered improved spontaneous alternation performance through Y-maze test assessed with Aβ(1–42)-infused mouse model. Collectively, while carprofen accelerates the conversion of Aβ monomers into fibrils in vitro, the drug ameliorates the major pathological hallmarks of AD in vivo.

## Introduction

Alzheimer’s disease (AD) is the most common neurodegenerative disorder with its pathogenesis driven by the formation of amyloid-β (Aβ) plaques in the brain^1,2^. Amyloid plaques are assembled from Aβ monomers followed by a seeding-nucleation process to produce several intermediates, such as soluble Aβ oligomers^3,4^. Overproduced Aβ aggregates trigger cascades of pathologic events, such as post-synaptic degeneration, tau phosphorylation, and neuroinflammation in AD^5,6^. The oligomers then elongate into insoluble fibrils, eventually leading to neuronal apoptosis and cell death^7^. As Aβ protein is the core constituent of AD progression, intensive efforts to mitigate or prevent its aggregation have been attempted in the hopes of developing a potential therapy for AD^8^.

In this study, we observed the effect of carprofen, a non-steroidal anti-inflammatory drug (NSAID), on Aβ deposition utilizing thioflavin T (ThT) fluorescence assay, which measures the amount of β-sheet complex of Aβ aggregates^9^, and found carprofen accelerating the formation of Aβ fibrils. We further assessed in vivo study after intraperitoneally injecting carprofen (25 mg/kg/day) to a female 5XFAD (B6SJL-Tg(APPSwFlLon, PSEN1*M146L*L286V) 6799Vas/Mmjax) transgenic mouse model of AD followed by staining for Aβ plaques utilizing thioflavin S (ThS) dye and detecting reactive astrocytes by glial fibrillary acidic protein (GFAP) antibody. Resultantly, we examined carprofen-treated mice brains displaying a significant increase in plaque levels but decreased burden of reactive astrocytes compared to vehicle-treated 5XFAD mice brains. To determine whether carprofen affects the development of pathological responses induced by Aβ aggregation, we lysed the cortical and hippocampus brain lysates and examined for possible alterations in the expression levels of reactive astrocytes, activated microglia, pre- and post-synaptic density proteins, and phosphorylated tau. Furthermore, we utilized an Aβ(1–42)-infused mouse model to evaluate if carprofen rescues cognitive and behavioral deficits through the assessment of Y-maze test. By examining the effects upon carprofen treatment on the aforementioned pathological parameters, our results exposed the ability of carprofen to target the key Alzheimer-like phenotypes by alleviating the deficits induced concomitant to Aβ aggregation.

## Results

### Carprofen accelerates Aβ aggregation but decreases reactive astrogliosis in 5XFAD mice brains

**Figure 1:**
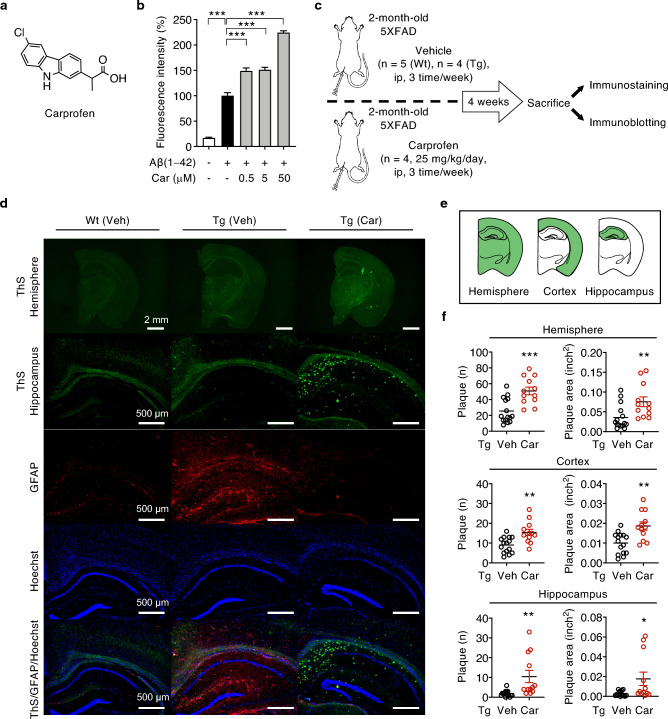
Carprofen accelerates Aβ fibrillization and increases Aβ plaque levels but decreased GFAP levels in 5XFAD mice brains. (**a**) Chemical structure of carprofen. (**b**) ThT assay exhibiting accelerated Aβ aggregation after three days of carprofen (0.5, 5, and 50 μM) incubation with monomeric Aβ(1–42) (50 μM) at 37 °C. The intensity levels were normalized to Aβ aggregates (100%, 3 days). (**c**) Timeline of in vivo experiment. (**d**) Representative immunostained hemisphere and hippocampus images of wild-type and 5XFAD transgenic mice after the administration of the vehicle (wild-type, n = 5; transgenic, n = 4) or carprofen (25 mg/kg/day, n = 4). Aβ plaques were stained with ThS (green), astrocytosis levels examined using GFAP antibody (red), and nuclear staining by Hoechst (blue). Scale bars = 2 mm (upper, hemisphere) and 500 μm (bottom, hippocampus). All hemisphere and hippocampus brain images are presented in the Supplementary Figs. S1 and S2, respectively. (**e**) Brain hemisphere, cortical, and hippocampal regions assessed for Aβ plaque measurements. (**f**) Quantitative measurements of Aβ plaque and area in hemisphere, cortical, and hippocampal brain regions after vehicle (black circle) or carprofen (red circle) treatment. Three consecutive brain sections were stained and quantified for each mouse. The data represent the mean ± SEM and the statistical analyses were performed by one-way analyses of variance (ThT data) and two-tailed unpaired *t*-test (plaque number and area densitometry) followed by Bonferroni’s post-hoc comparisons tests (*P < 0.05, **P < 0.01, ***P < 0.001, other comparisons not significant). Wt, wild-type; Tg, transgenic; ip, intraperitoneal; Veh, vehicle; Car, carprofen; ThS, thioflavin S.

To investigate the inhibitory effect of carprofen (Fig. 1a) on Aβ aggregation, the drug (0.5, 5, and 50 μM) was incubated with Aβ(1–42) monomers (50 μM) for 3 days at 37 °C and observed by ThT assay, a commonly used probe of amyloid fibrils^9^. Results showed dose-dependent increment of fluorescence intensity upon carprofen treatment, exhibiting the highest concentration at 50 μM (Fig. 1b), implying the addition of carprofen allows for significant acceleration of Aβ fibrillization.

Although higher plaque deposition is reported among female 5XFAD transgenic mice compared to male mice of the same age^10,11^, both male and female 5XFAD mice develops plaques accompanied by gliosis as young as two months of age^11,12^. Therefore, utilizing a 2-month-old female 5XFAD transgenic mice, we further examined the therapeutic function of carprofen in vivo (Fig. 1c). Carprofen (25 mg/kg/day, n = 4) or vehicle (n = 4) was administered by three intraperitoneal injections each week for a total of 4 weeks. Vehicle was also injected to age-matched wild-type (n = 5) as a control. We then sacrificed all mice groups and extracted their brains to primarily observe the change in Aβ plaque levels through immunohistochemical staining. Three consecutive brain sections were acquired and stained for each mouse to quantify the change in total plaque and area between carprofen- and vehicle-treated groups. Consistent with in vitro result, mice brains of carprofen-treated displayed greater number and larger size of Aβ plaques in both the cortical and hippocampal regions in comparison to the brains of vehicle-treated mice (Fig. 1d–f). Furthermore, we observed Aβ-associated inflammatory response, astrogliosis, upon carprofen treatment through histochemical imaging of reactive astrocyte levels by GFAP antibody. Compared to vehicle-treated transgenic mice brains, lowered GFAP expressions have been detected in the brains of carprofen-treated mice (Fig. 1d), implying carprofen can ameliorate astrogliosis while accelerating the aggregation of Aβ peptides into β-sheet-rich fibrils, elevating plaque levels. Altogether, the administration of carprofen induces contrasting levels of plaques and reactive astrocytes in young brains of 5XFAD mice.

### Carprofen alleviated the expression levels of AD-related pathological features

**Figure 2:**
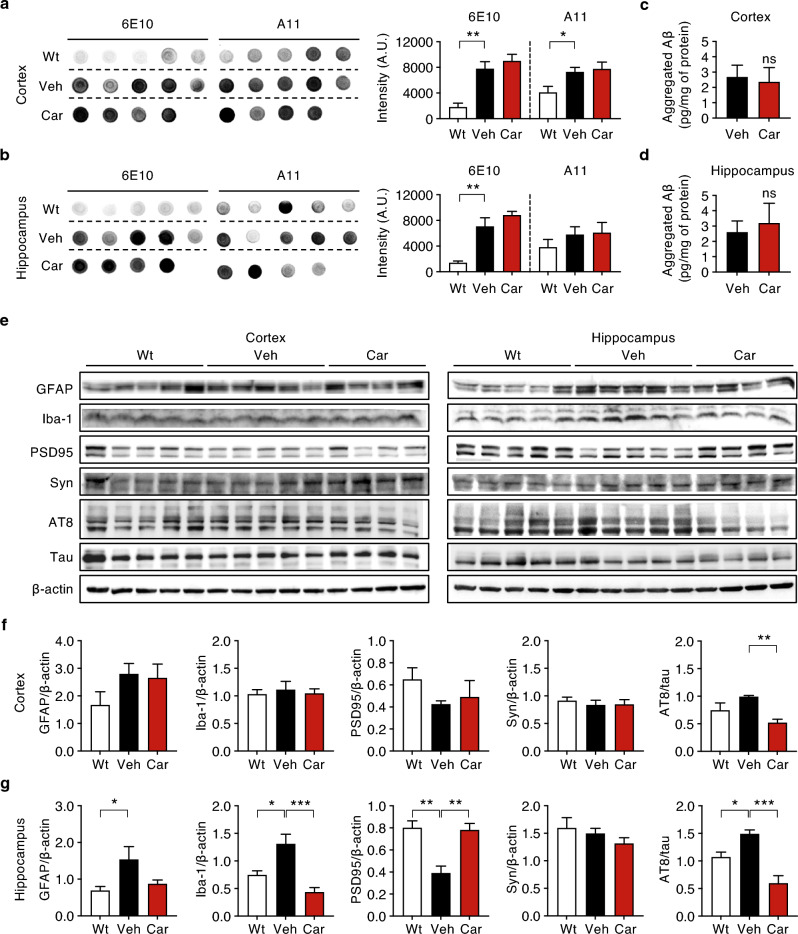
Carprofen affects the expression levels of key Alzheimer-like biomarkers involved in Aβ aggregation. (**a**, **b**) Dot blots and densitometry of the cortical and hippocampal regions to quantify the total soluble Aβ and oligomer concentrations by utilizing anti-Aβ 6E10 antibody and anti-oligomer A11 antibody, respectively. (**c**, **d**) The aggregated Aβ levels of vehicle- and carprofen-treated groups in both the cortex and hippocampus after ELISA. (**e–g**) Western blot and densitometry of Alzheimer-related biomarkers in (**f**) cortex and (**g**) hippocampus brain lysates. The densitometry data analyzed the levels of GFAP, Iba-1, PSD95, synaptophysin, and phosphorylated tau expressions. The original dot blots and full-membrane results with their respective β-actins are presented in Supplementary Fig. S3. All data represent the mean ± SEM and the statistical analyses were performed by one-way analysis of variance excluding ELISA data using two-tailed unpaired *t*-test with the comparison to the vehicle-treated 5XFAD mice group (Veh, black) (*P < 0.05, **P < 0.01, ***P < 0.001, other comparisons not significant). Wt, wild-type; Veh, vehicle; Car, carprofen; GFAP, glial fibrillary acidic protein; Iba-1, ionized calcium-binding adaptor molecule 1; PSD95, post-synaptic density protein 95; Syn, synaptophysin; AT8, phosphorylated tau (S202, T205).

Both in vitro and in vivo results showed increased levels of Aβ fibrils and plaques, suggesting carprofen induces an increment of insoluble Aβ forms. Such result raised the question of whether carprofen could also advocate the concentration of soluble Aβ forms, such as Aβ oligomers. We performed dot blot assay to quantify the concentrations of total soluble Aβ and oligomers using 6E10 and A11 antibodies, 6E10 detecting all Aβ forms while A11 detecting oligomeric Aβ in soluble fractions. Resultantly, we found a slight increment of total soluble Aβ and oligomer concentrations in both the cortical and hippocampal brain lysates of carprofen-treated mice compared to vehicle-treated mice (Fig. 2a, b). However, the increasing tendency was not to the extend to show significance. To clearly examine the mechanism of action of carprofen on aggregated Aβ levels, we assessed enzyme-linked immunosorbent assay (ELISA) and found no significant change in the aggregated Aβ load among carprofen-treated group compared to vehicle-treated group in both the cortex and hippocampus (Fig. 2c, d). Such findings suggest carprofen does not show direct interaction with oligomeric Aβ. Thus, we further assessed western blot to investigate whether carprofen alters the expression levels of AD pathological features (Fig. 2e–g).

As a NSAID drug, we first examined the inflammatory responses through the evaluation of astrogliosis and microgliosis upon carprofen treatment by detecting GFAP and ionized calcium-binding adaptor molecule 1 (Iba-1) antibodies, respectively. Although decreased levels of GFAP and Iba-1 expressions in the cortex of carprofen-treated mice was not enough to show significance, we observed carprofen treatment lowering the levels of GFAP and Iba-1 in both cortex and hippocampus (Fig. 2f, g). The inhibitory function of carprofen in the activation of resident astrocyte and microglia levels implies the capability of carprofen to prevent neuroinflammatory responses. In fact, the hippocampal astrocytes exposed to Aβ typically instigate Aβ-induced dysfunction of *N*-methyl D-aspartate receptors, disrupting neuron-glial signal transmission that creates consequences on the synaptic transmission and plasticity^13^. Since carprofen prevented the inflammatory responses in the hippocampus, we conducted further investigations to examine if carprofen treatment could ameliorate synaptic dysfunction.

The change in pre- and post-synaptic density proteins, which are associated with synaptic plasticity and memory deficits, were investigated by the expression levels of synaptophysin and post-synaptic density protein 95 (PSD95), respectively. The pre-synaptic biomarker indicated by synaptophysin was unaffected in the cortex but decreased in the hippocampus of carprofen-treated mice group. On the other hand, the PSD95 expressions were upregulated in both the cortex and hippocampus after the treatment of carprofen compared to vehicle-treated mice. Consistent to the effect of GFAP expression levels, carprofen induced significant restoration of PSD95 expressions in both the cortex and hippocampus, although the altered level in the cortex was not enough to show significance. Considering synapses are the earliest site of pathology^14^, our finding demonstrates carprofen could restore synaptic dysfunction when administered at the age when AD pathology initiates in 5XFAD mouse model.

In addition to the presence of Aβ aggregates, phosphorylated tau is another key pathological feature of AD with tau phosphorylation inducing pathological mechanisms, including synaptic impairment^15^. As we observed ameliorated Aβ-induced synaptic dysfunction upon carprofen treatment, we also examined phosphorylated-tau (p-tau) burden to evaluate the ability of carprofen against tau pathology. When measuring p-tau expression by AT8 antibody, which recognizes the phosphorylation sites S202 and T205 in tau, we discovered significant decrease in p-tau levels among carprofen-treated mice (Fig. 2f, g), indicating carprofen is sufficient to inhibit the build-up of p-tau proteins. Altogether, our western blot results demonstrated the treatment of carprofen alleviating the activation of reactive astrocytes, microglia, and neuronal synaptic dysfunction, in addition to inhibiting hyperphosphorylation of tau proteins.

### Carprofen improves cognitive behavioral performance on the Y-maze task utilizing Aβ(1–42)-infused mice

The western blot results revealed a significant increase in PSD95 expression levels in the hippocampus upon carprofen treatment, implying the drug could alleviate synaptic dysfunction. As synapse failures induced by Aβ oligomers are the result of memory loss, the improvement in post-synaptic proteins by carprofen raises the question of whether carprofen could also improve early memory deficits. To observe the therapeutic effect of carprofen on spatial working and hippocampal memory declines, we performed Y-maze test utilizing an Aβ(1–42)-infused mouse model with Alzheimer-like cognitive impairments^16^.

Given the potent effect of soluble oligomeric Aβ on the impairment of learning and memory^17^, we sought to examine the incubation period required for carprofen to regulate the burden of Aβ oligomeric forms. Based on the ThT assays, we identified the concentrations (0.5 μM, 5 μM, and 50 μM) of carprofen that significantly influenced the Aβ aggregation process. In this regard, among the three concentration levels, we investigated if the lowest (0.5 μM) and highest (50 μM) doses of carprofen could alter Aβ oligomeric levels. As a result, through dot blot assay, we discovered 2-day incubated pre-treated samples of both low and high concentrations of carprofen added with Aβ(1–42) monomer exhibiting decreased Aβ oligomeric levels compared to 2-day Aβ(1–42) aggregates-treated sample (Supplementary Fig. S4). Thus, we used 2-day incubation for our behavior study.

**Figure 3:**
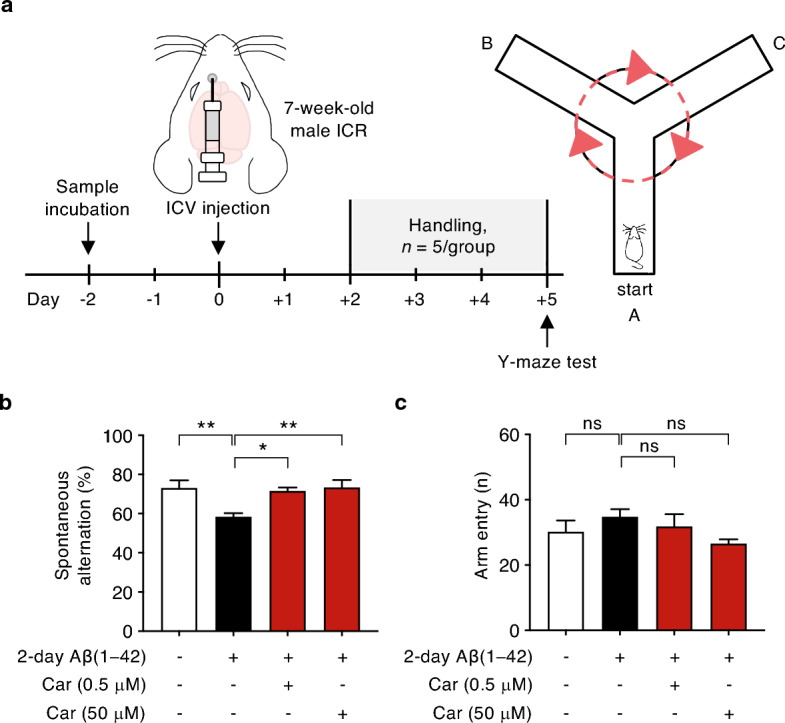
Improvement of spontaneous alternation performance on Y-maze task using an Aβ(1–42)-infused mouse model injected with carprofen co-incubated with Aβ(1–42) monomer by ICV. (**a**) The overall experimental scheme displaying the time course of sample incubation followed by the schematic brain region to show the ICV injection site of ICR mice (male, 7-week-old, n = 5/group) to create an Aβ(1–42)-infused mouse model, with reduced cognitive behavioral performance, for a Y-maze test. (**b**) The percentage of spontaneous alternations and (**c**) the number of entries observed on four Aβ(1–42)-infused ICR mice groups. The prepared groups are as following: vehicle (white, n = 5), Aβ(1–42) aggregates (0.05 nmole in PBS, black, n = 5), in addition to carprofen (0.5 or 50 μM) co-treated with Aβ(1–42) monomer (0.05 nmole in PBS, red, n = 5/group), All data represent the mean ± SEM and the statistical analyses were performed by one-way analysis of variance with the comparison to 2-day Aβ(1– 42) aggregates-treated group (black) (*P < 0.05, **P < 0.01, ***P < 0.001, other comparisons not significant). ICV, intracerebroventricular; ICR, imprinting Control Region; Car, carprofen.

Based on the previous study demonstrating a single intracerebroventricular (ICV) injection of Aβ(1–42) enforcing cognitive impairments^16,18^, four pre-treated samples, which are vehicle, Aβ(1–42) aggregates (0.05 nmole in PBS), and Aβ(1–42) monomer (0.05 nmole in PBS) co-treated with two concentrations of carprofen (0.5 or 50 μM), were prepared by 2-day incubation at 37 °C. Afterwards, 5 μL of each incubated sample has been injected into the ICV region of an ICR mice (male, 7-week-old, n = 5/group), and subjected for Y-maze study (Fig. 3a). The vehicle and Aβ(1–42) aggregates-treated mice groups were prepared as controls. Cognitive ability of short-term spatial working memory was measured by the spontaneous alternation of each mouse on the Y-maze. Resultantly, we found carprofen-treated (0.5 and 50 μM) groups (red) with significant enhanced spontaneous alternation rate, compared to 2-day Aβ(1–42) aggregates-treated control group (black) (Fig. 3b). The exploratory activity observed by the total arm entries showed no significant differences among the groups (Fig. 3c). Consistent with the western blot results displaying alleviation of pathological parameters significantly in the hippocampus, we found both low (0.5 μM) and high (50 μM) dosage of carprofen treatment rescuing impaired hippocampal-dependent memory through Y-maze behavior test.

**Figure 4:**
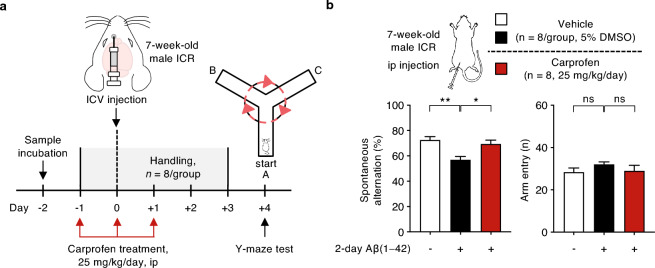
Improvement of spontaneous alternation performance on Y-maze task using an Aβ(1–42)-infused mouse model injected with carprofen co-incubated with Aβ(1–42) monomer by peripheral administration. (**a**) The experimental scheme displaying the time course of sample incubation followed by the schematic brain region to show the ICV injection site of ICR mice (male, 7-week-old, n = 8/group) to create an Aβ(1 – 42)-infused mouse model, with reduced cognitive behavioral performance, followed by intraperitoneal injections of carprofen in prior to Y-maze test. (**b**) The percentage of spontaneous alternations and the total number of entries observed on Aβ(1 – 42)-infused ICR mice groups. The prepared groups are as following: vehicle (white, n = 8), 2-day Aβ(1– 42) aggregates (0.05 nmole in PBS, black, n = 8), and a second 2-day Aβ(1–42) aggregates group with three daily intraperitoneal injections of carprofen (25 mg/kg/day, red, n = 8). All data represent the mean ± SEM and the statistical analyses were performed by one-way analysis of variance with the comparison to 2-day Aβ(1 − 42) aggregates-treated group (black) (*P < 0.05, **P < 0.01, ***P < 0.001, other comparisons not significant). ICV, intracerebroventricular; ICR, imprinting control region; ip, intraperitoneal.

For further insight on the therapeutic effects of carprofen, we performed an additional Y-maze study in which carprofen has been administered peripherally. For this round of Y-maze study, two Aβ samples, vehicle and Aβ(1–42) aggregates (0.05 nmole in PBS), were prepared by 2-day incubation at 37 °C. Subsequently, 5 μL of the incubated sample has been injected into the ICV region of an ICR mice (male, 7-week-old, n = 8/group). In specific, a total of three mice groups were prepared as following: vehicle-treated (white, n = 8), 2-day Aβ(1–42) aggregates-treated (black, n = 8), and a second 2-day Aβ(1–42) aggregates-treated group with three daily intraperitoneal injections of carprofen (25 mg/kg/day, red, n = 8). We then measured the spontaneous alternation behavior of each mouse on the Y-maze (Fig. 4a). As a result, we found significant alleviation on the alternation rate upon carprofen-treated group (red) compared to 2-day Aβ(1–42) aggregates-treated group (black), meanwhile the total number of entries did not show significant difference among the groups (Fig. 4b). Consistent to the first Y-maze study with carprofen injected directly into the ICV region, a peripheral administration of carprofen by intraperitoneal injection was found to be also effective in mitigating cognitive impairments via enhanced spontaneous alternation behavior.

### The effect of carprofen does not induce any pathological change on wild-type mice

**Figure 5:**
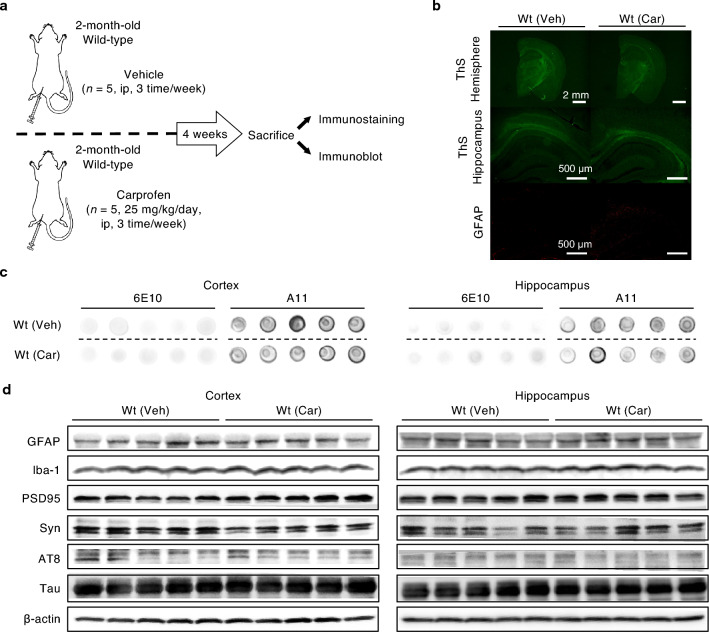
Carprofen does not induce any significant change in the expression levels of Alzheimer-like characteristics among non-AD mice. (**a**) Timeline of in vivo experiment utilizing wild-type animals. (b) Representative immunostained hemisphere and hippocampus images of wild-type brains after the administration of the vehicle (n = 5) or carprofen (25 mg/kg/day, n = 5). Scale bars = 2 mm (upper, hemisphere) and 500 μm (bottom, hippocampus). (**c**) Dot blot on total soluble Aβ and oligomer concentrations by utilizing anti-Aβ antibody 6E10 and anti-oligomer antibody A11, respectively, in the cortex and hippocampus. (**d**) Western blot of Alzheimer-like biomarkers in cortex and hippocampus brain lysates. All hemisphere and hippocampus brain images and original dot blot membranes are shown in Supplementary Fig. S5. In addition to the full-membranes and their respective β-actins along with the densitometry analyses on the levels of GFAP, Iba-1, PSD95, synaptophysin, and phosphorylated tau expressions are presented in Supplementary Fig. S6. Wt, wild-type; Veh, vehicle; Car, carprofen; GFAP, glial fibrillary acidic protein; Iba-1, ionized calcium-binding adaptor molecule 1; PSD95, post-synaptic density protein 95; Syn, synaptophysin; AT8, phosphorylated tau (S202, T205).

To clearly understand the potential effect of carprofen, we also examined whether carprofen shows alteration in Alzheimer-like features when administered to non-AD mice. Consistent to the in vivo study performed on transgenic mice, female wild-type animals were either treated with vehicle (n = 5) or carprofen (25 mg/kg/day, n = 5) by intraperitoneal injection three times a week for a total of four weeks (Fig. 5a).

After the administration, we assessed immunohistochemical staining to justify if carprofen endorses potential effect to the brains of wild-type mice. As expected, no plaques or GFAP expression were observed in both wild-type groups, implying the treatment of carprofen does not affect non-AD mice brains (Fig. 5b). Further, we determined whether carprofen induced change in the concentrations of total soluble Aβ and oligomers in wild-type mice through dot blot analyses with 6E10 and A11 antibodies (Fig. 5c). In both the cortex and hippocampus, no significant change was found in the levels of soluble Aβ nor oligomer concentrations.

As we discovered alleviated results upon carprofen treatment to 5XFAD transgenic mouse model, we used western blots to observe for potential alterations in all the aforementioned AD-related biomarkers upon carprofen treatment to wild-type mice (Fig. 5d). Contrast to the ameliorating responses observed in 5XFAD mice, carprofen did not induce any changes when treated to wild-type mice. In fact, the intensity of the inflammatory, synaptic, p-tau expression levels reflected on both the cortical and hippocampal brain lysates of carprofen-treated wild-type group were similar to the signal shown on vehicle-treated wild-type group. These results suggest carprofen shows therapeutic efficacy by ameliorating the pathological features of AD when treated to 5XFAD transgenic mice.

## Discussion

In this study, we report the ability of carprofen, a NSAID, that interferes with Aβ deposition process by alleviating the key AD pathological pathways disrupted in parallel to Aβ aggregation. Increased Aβ fibrillar levels with elevated Aβ plaques in carprofen-treated mice brains were observed while carprofen attenuated Alzheimer-like phenotypes, such as reducing the activation of reactive astrocytes and microglia, recovering post-synaptic dysfunction, inhibiting further phosphorylation of tau, and rescuing working memory deficit. Although the major target of carprofen is currently not available to be used in humans^19,20^, the effects found upon carprofen treatment from our findings support to warrant NSAID as an essential drug type to potentially influence the pathological hallmarks endorsed by the aggregation of Aβ. However, based on our findings, we observed regional differences among carprofen-treated mice as the effects of carprofen, including reactive astrocytes and microglia and PSD95 expressions, showed statistical significance predominantly in the hippocampus. Since our in vivo study has a small sample size, this limitation could be the potential reason for the non-significant findings shown in the cortical regions of our western blot. Thus, larger sample size with long-term administration period should be considered in addition to future studies evaluating the mechanism of carprofen within various brain regions to determine if carprofen shows regional differences in delivering and distributing its effects.

The beneficial action of NSAIDs is by the inhibition of two isoforms of cyclooxygenase (COX) enzyme, COX-1, and COX-2, which allows NSAIDs to reduce the biosynthesis of pro-inflammatory prostaglandins^21,22^. However, the exact mechanism of action of COX-inhibiting NSAIDs is not yet fully understood, proposing the therapeutic effects may not be related to COX inhibition^23^. In this study, we observed an improvement in spontaneous alternation behavior upon the treatment of carprofen, a NSAID with a mechanism of action based on COX inhibition pathway^24^, warranting the possibility of COX-inhibiting drugs to ameliorate impaired working memory and spatial learning ability. Although an Aβ(1–42)-infused mouse model mimics AD-like phenotypes, it may not fully represent the complexity of AD, thus, behavioral studies using AD transgenic mouse models could be informative to further validate our findings. Moreover, as a Y-maze test evaluates hippocampus-dependent short-term spatial working memory^25^, further studies are necessary to explore the potential of COX inhibition in later stages of AD progression to show effectiveness in treating long-term memory deficits.

Collectively, previous reports on other NSAIDs, including ibuprofen, naproxen, and indomethacin, exhibited decrement of Aβ levels and improved cognitive function in AD mouse models^26–28^. Although carprofen treatment led to an increase in plaque burden, consistent to other NSAIDs, we observed improvement in key Alzheimer-like phenotypes in vivo (Fig. 2e–g), as evidenced by enhanced spontaneous alternation rates in Y-maze behavior tests (Figs. 3 and 4). Moreover, our dot blot and ELISA assays (Fig. 2a–d) revealed that carprofen does not directly interact with the formation of oligomeric Aβ form. Instead, our in vitro results suggest carprofen may have enhanced the conversion of Aβ oligomers into mature fibrils, thereby increasing the aggregation rate while reducing the concentration of toxic oligomeric species. This process could have interrupted the toxicity induced by prominent AD mechanisms, such as neurotoxicity, synaptic impairment, and cognitive decline^29–31^. Further studies specifying a clear mechanism of how carprofen interacts with Aβ would not only clarify by which the drug advocates to relieve Alzheimer-like phenotypes, but also provide evidence warranting NSAID as a rational drug type for future AD treatment.

## Methods

### Animals

5XFAD transgenic mice (B6SJL-Tg(APPSwFlLon, PSEN1*M146L*L286V) 6799Vas/Mmjax) and wild-type (B6SJL) mice were obtained from Jackson laboratory (USA). For the assessment of spatial learning and memory by Y-maze test, ICR mice (male, 7-week-old) were purchased from Orient Bio Inc. (Seoul, Korea). All mice were maintained in groups of 4–5/cage in the animal facility of Yonsei University (Seoul, Korea). The mice were bred under controlled temperature and humidity given with 12:12 hour light–dark cycle and given ad libitum access to food and water.

All animal experiments were carried out in accordance with the National Institutes of Health guide for the care, use of laboratory animals (NIH Publications), and the ARRIVE guidelines. The animal experiment protocols were also approved by the Animal Institutional Animal Care and Use Committee of Yonsei University (Seoul, Korea, IACUC-202103-1221-01).

### ThT fluorescence assays

Synthetic Aβ(1–42) peptides were dissolved in DMSO as 1 mM stock, and the carprofen stock was prepared at 10 mM in DMSO. To investigate the ability of carprofen to inhibit the aggregation of Aβ, 50 μM of Aβ(1–42) peptides were incubated with three different concentrations of carprofen (0.5, 5, and 50 μM) for 3 days at 37 °C, at a final DMSO concentration of 5% DMSO in deionized water.

After the incubation period, the samples were analyzed by ThT fluorescence assays to quantify the level of β-sheet complex of Aβ aggregates. 75 μL of ThT solution concentration (5 μM in 50 mM glycine buffer, pH 8.5) and 25 μL of the incubated samples (1:3 sample/ThT solution, v/v) were loaded in a 96-well half area black microplate (3694, Corning Inc., USA). Fluorescence intensity of Aβ-bound ThT (Sigma-Aldrich, USA) was measured at 450 nm (excitation) and 485 nm (emission) by using a microplate reader (Infinite M200 Pro, Tecan, USA).

### Drug administration

5XFAD mice (female, n = 4–5/group) received vehicle (5% DMSO/5% Tween 80 in PBS, n = 5) or carprofen (25 mg/kg/day, 5% DMSO/5% Tween 80 in PBS, n = 5) via intraperitoneal injection three times per week for a total of 4 weeks. Age-matched wild-type mice (5% DMSO/5% Tween 80 in PBS, n = 5) were used as controls. For the wild-type in vivo set, wild-type mice (female, n = 5/group) were treated with vehicle (5% DMSO/5% Tween 80 in PBS, n = 5) or carprofen (25 mg/kg/day, 5% DMSO/5% Tween 80 in PBS, n = 5) via intraperitoneal injection for three times a week for a total of 4 weeks. After four weeks of administration, all mice were anesthetized by 4% avertin via intraperitoneal injection and sacrificed for immunohistochemical or immunoblotting analyses.

### Histochemistry of brain sections

Three consecutive mice brains per each mouse were extracted for each mouse hemisphere fixed with 4% paraformaldehyde for 24 hours and immersed in 30% sucrose for 48 hours before cryosection. The other hemisphere was dissected to obtain cortex and hippocampus lysates. Fixed brain tissues were sliced with 35-μm thickness and detected the mice brains for reactive astrocytes by GFAP antibody (abcam, USA) followed by staining with 500 μM of ThS (Sigma-Aldrich, USA) dissolved in 50% ethanol for seven minutes in the dark for Aβ plaque visualization. The brain tissues were then destained with 100%, 90%, and 70% ethanol solutions for one minute each and washed twice in PBS for one minute each. Tissue images were obtained by a fluorescence microscope (Leica DM2500) with a LAS X software program. The number and area of Aβ plaques in cortex and hippocampus was analyzed by Image-J software.

### ELISA

To quantify aggregated Aβ in soluble fraction of cortical and hippocampal lysates, 10 μg of protein was used for analysis. The assay was conducted according to the instructions of the aggregated Aβ ELISA kit (Invitrogen, USA) followed by measuring the absorbance (450 nm) utilizing the SpectraMax M2 microplate reader (Molecular Devices, USA).

### Immunoblotting

The cortical and hippocampal regions were each collected from the brains of the experimented 5XFAD and wild-type mice. The brain tissues were homogenized in RIPA buffer consist of protease and phosphatase inhibitors, then incubated on ice for 30 minutes, followed by centrifuging at 14,000 rpm, at 4 °C for additional 30 minutes. The protein concentrations in supernatants were quantified using Pierce™ BCA protein assay kit (Thermo Fisher Scientific, USA).

Dot blots were performed to measure the total soluble Aβ and oligomer concentrations with anti-Aβ antibody 6E10 (1:2,000, host: mouse, Biolegend, USA) and anti-oligomer antibody A11 (1:2,000, host: rabbit, Invitrogen, USA), respectively. 20 μg of cortex and 10 μg of hippocampus lysates were loaded on a nitrocellulose membrane and dried for 30 minutes.

For western blot analysis, 25 μg of brain lysates were loaded to SDS-PAGE gel and transferred to nitrocellulose membrane. The membranes were incubated with anti-GFAP (1:2,000, host: chicken, Millipore Corporation, USA), anti-iba1 (1:2,000, host: mouse, Millipore Corporation, USA), anti-PSD (1:1,000, host: mouse, Invitrogen, USA), anti-synaptophysin (1:1,000, host: mouse, Millipore Corporation, USA), anti-phospho-tau (Ser202, Thr205, AT8, 1:1,000, 3% BSA, host: mouse, Invitrogen, USA), anti-tau (1:1,000, host: rabbit, abcam, USA), and anti-β-actin (1:2,000, host: mouse, Millipore Corporation, USA) overnight at 4 °C. The membranes were then detected by HRP-conjugated secondary antibodies: anti-chicken (1:20,000, Jackson ImmunoResearch, USA), goat anti-mouse (1:20,000, Bethyl Laboratories, USA), and goat anti-rabbit (1:10,000, Jackson, USA). The protein levels for each mice group were quantified by densitometry using Image-J software.

### Preparation of Aβ(1–42)-infused mouse model by Aβ and carprofen injection

Since soluble oligomeric Aβ is the potent factor that directly impairs learning and memory abilities, the effect of carprofen in regulating Aβ oligomeric forms was evaluated by assessing two Y-maze studies. Prior to behavior study, both low (0.5 μM) and high (50 μM) concentrations of carprofen pre-treated with Aβ(1–42) monomer incubated along with Aβ(1–42) aggregates only sample incubated at various time periods (0-day, 1-day, 2-day, and 3-day). All samples were spotted on the nitrocellulose membrane to detect for possible change in soluble oligomeric concentrations via dot blot assay utilizing A11 antibody. As a result, 2-day incubated pre-treated samples of both low (0.5 μM) and high (50 μM) concentrations of carprofen treated with Aβ(1–42) monomer exhibited decreased Aβ oligomeric forms compared to 2-day Aβ(1–42) aggregates-treated sample, thus 2-day incubation condition has been used for the study.

The first Y-maze study used male ICR mice (n = 20) which were randomly divided into four groups (n = 5/group) and habituated for 2 days. A total of four samples, either pre-treated with or without carprofen, were incubated at 37 °C for 2 days. The four samples are as following: vehicle (5% DMSO), Aβ(1–42) aggregates (0.05 nmole in PBS, 5% DMSO), and Aβ(1–42) monomer (0.05 nmole in PBS) co-treated with 0.5 or 50 μM of carprofen (0.05 nmole in PBS, 5% DMSO). Since previous reports showed that a single ICV injection of Aβ(1–42) monomers enforces cognitive impairments^16,18^, 5 μL of each sample was injected via ICV region of an ICR mice (male, 7-week-old, n = 5/group) using a Hamilton syringe with a 26-gauge needle. The vehicle and Aβ(1–42) aggregates-treated groups were prepared as controls.

For the second round of Y-maze task, another Aβ(1–42)-infused mouse model was prepared with carprofen treated via intraperitoneal injection to mimic peripheral administration. A total of three mice groups were prepared as following: vehicle-treated (5% DMSO, n = 8), and 2-day Aβ(1–42) aggregates-treated (0.05 nmole in PBS, 5% DMSO, n = 8), and another 2-day Aβ(1–42) aggregates-treated group with three intraperitoneal injections of carprofen (25 mg/kg/day, 5% DMSO/5% Tween 80 in PBS, n = 8). Consistent to the first behavior study, 5 μL of the incubated samples were injected into the ICV region of ICR mice brains (male, 7-week-old) and subjected for Y-maze study.

### Y-maze task of ICV injected mice

Y-maze tests were performed in all mice to assess cognitive changes of short-term spatial working memory (by spontaneous alternation) and exploratory activity (by the number of total arm entries). Y-maze is a Y-shaped maze (40 cm long, 10 cm wide, 12 cm high) with three symmetrical arms at a 120° angle from each other. Each mouse was placed at one arm to freely move through the maze during an 8-minute session. The number of arm entries and triads were recorded to calculate the alternation percentage. An entry of a mouse was considered when all four limbs were within the arm, passing the middle circle region.

### Statistical analyses

Statistical analyses were conducted with GraphPad Prism 9. As for the statistical analyses, the plaque number and area densitometry of immunohistochemical analysis followed by western blot analyses of in vivo study utilizing wild-type animals were performed using two-tailed unpaired *t*-test. Other analyses, including ThT assays, dot blot, and western blot analyses of 5XFAD transgenic mice groups, in addition to Y-maze alternation rate and total number of entries observed upon Aβ(1–42)-infused model used one-way analysis of variance. All data used represented the mean ± SEM in addition to Bonferroni’s post-hoc comparisons tests (*P < 0.05, **P < 0.01, ***P < 0.001, other comparisons not significant).

## Supplementary Information

Supplementary Figures.
