# Supplementary material for: Carprofen alleviates Alzheimer-like phenotypes of 5XFAD transgenic mice by targeting the pathological hallmarks induced by amyloid-β aggregation
Source: Sci Rep. 2023 Jul 5;13:10889. doi: 10.1038/s41598-023-36167-4 (PMC10323003; doi:10.1038/s41598-023-36167-4)
Supplement: Supplementary file 1 — Supplementary Figures. [file 41598_2023_36167_MOESM1_ESM.pdf]

Carprofen alleviates Alzheimer-like phenotypes of 5XFAD transgenic mice by targeting the pathological hallmarks induced by amyloid-β aggregation

Authors

Donghee Lee[1,2], Ji Eun Ryoo[1,2], Seungpyo Hong[1,3,4,5], Hye Yun Kim[1,2], and YoungSoo Kim[1,2,3,6]*

Affiliations

[1]Department of Pharmacy, College of Pharmacy, Yonsei University, Incheon 21983, Republic of Korea.

[2]Yonsei Institute of Pharmaceutical Sciences, College of Pharmacy, Yonsei University, Incheon 21983, Republic of Korea.

[3]Yonsei Frontier Lab, Yonsei University, Seoul 03722, Republic of Korea.

[4]Pharmaceutical Sciences Division, School of Pharmacy, University of Wisconsin-Madison, Madison, Wisconsin, USA

[5]Wisconsin Center for NanoBioSystems, University of Wisconsin-Madison, Madison, Wisconsin, USA

[6]Department of Integrative Biotechnology and Translational Medicine, Yonsei University, Incheon 21983, Republic of Korea.

***To whom correspondence should be addressed**

YoungSoo Kim; y.kim@yonsei.ac.kr; Tel.: +82-32-749-4523; Fax: +82-32-749-4105

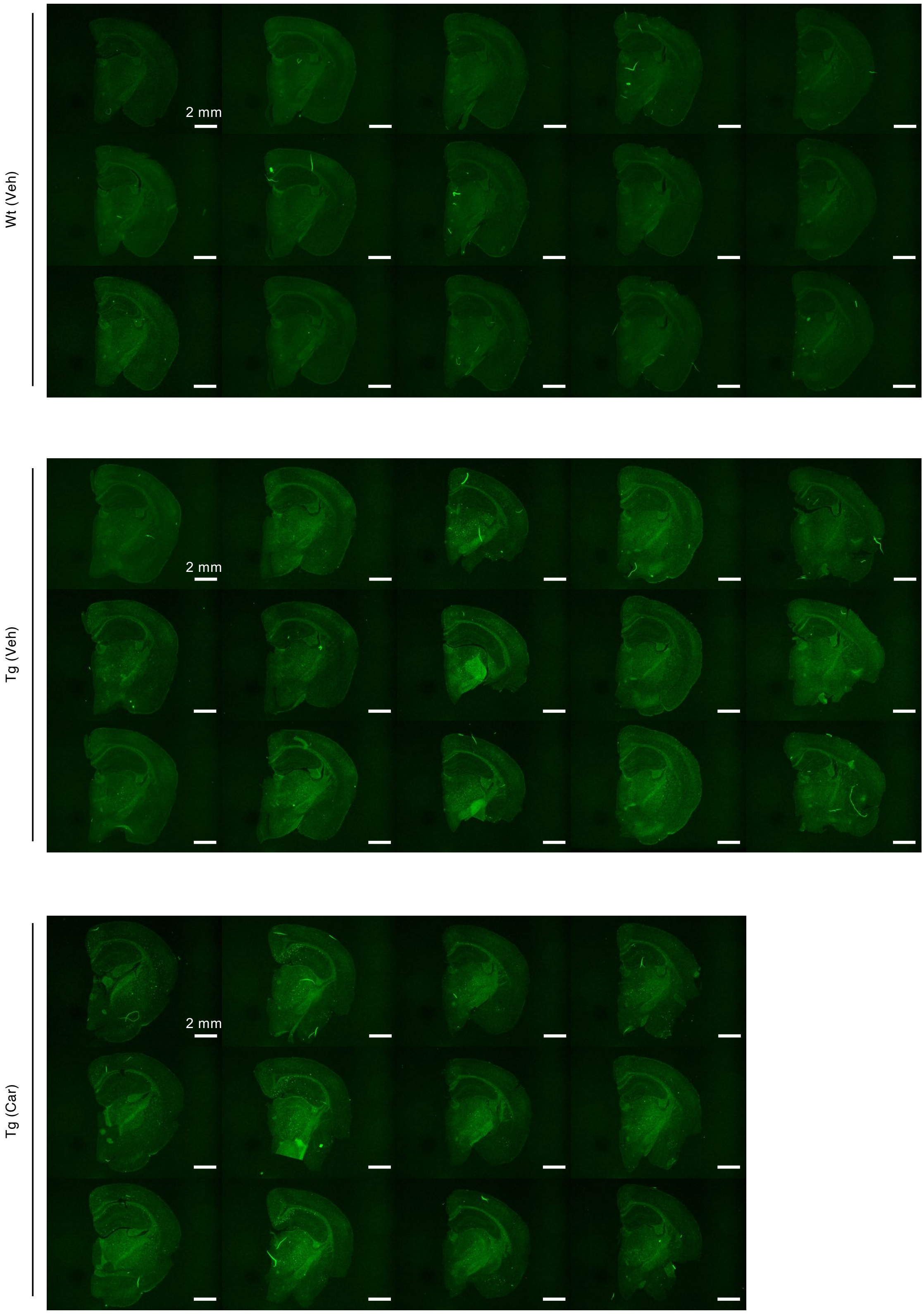

Supplementary Figure S1. Hemisphere images of wild-type and 5XFAD mice brains of all 3-sets of vehicle- and carprofen-treated groups. Wt: Wild-type; Tg: Transgenic; Veh: Vehicle; Car: Carprofen. Scale bar = 2 mm.

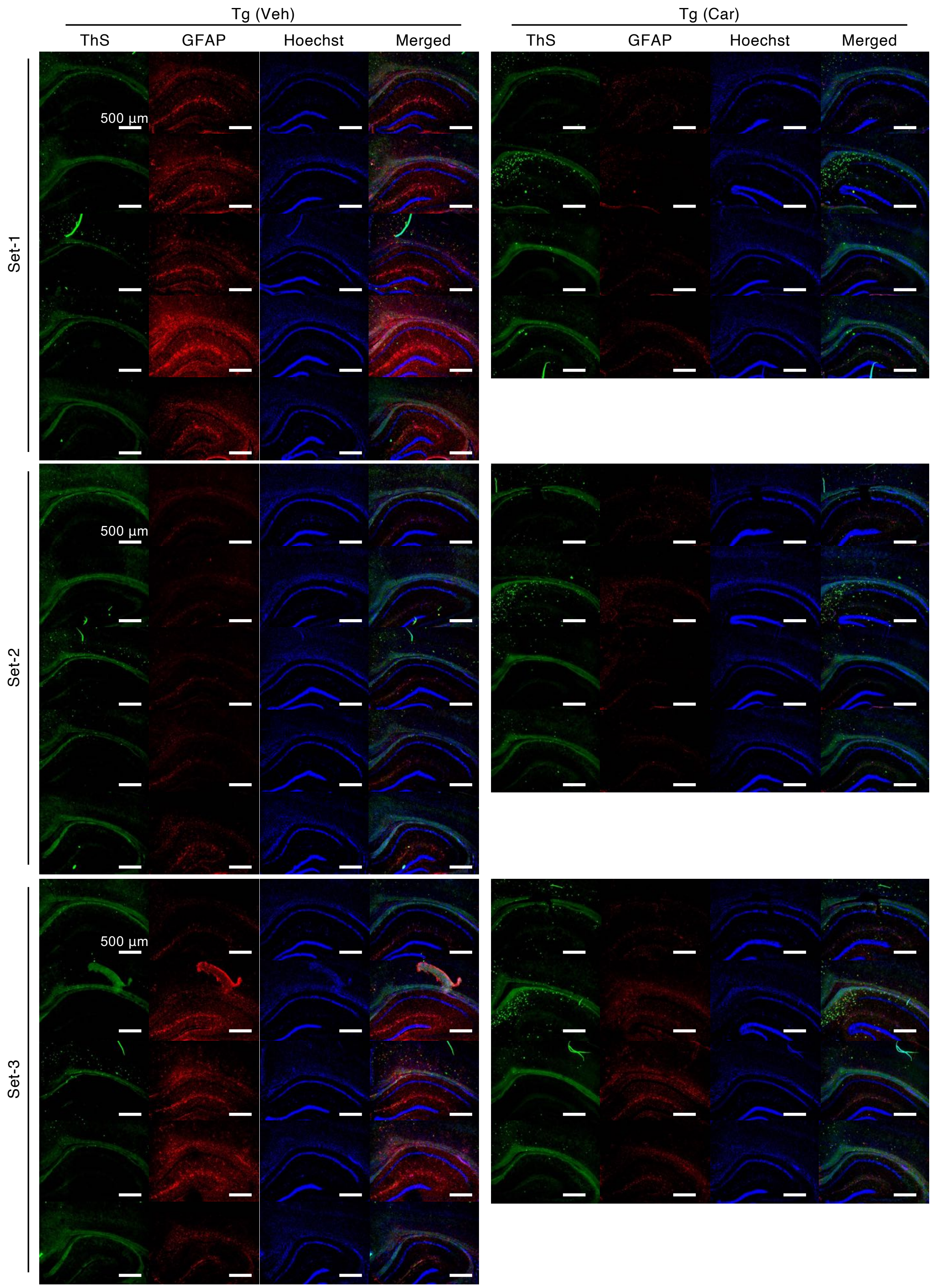

Supplementary Figure S2. Hippocampus images of 2.9-month-old wild-type and 5XFAD mice brains of all 3-sets of vehicle- and carprofen-treated groups. Wt: Wild-type; Tg: Transgenic; Veh: Vehicle; Car: Carprofen. Scale bar = 500 µm.

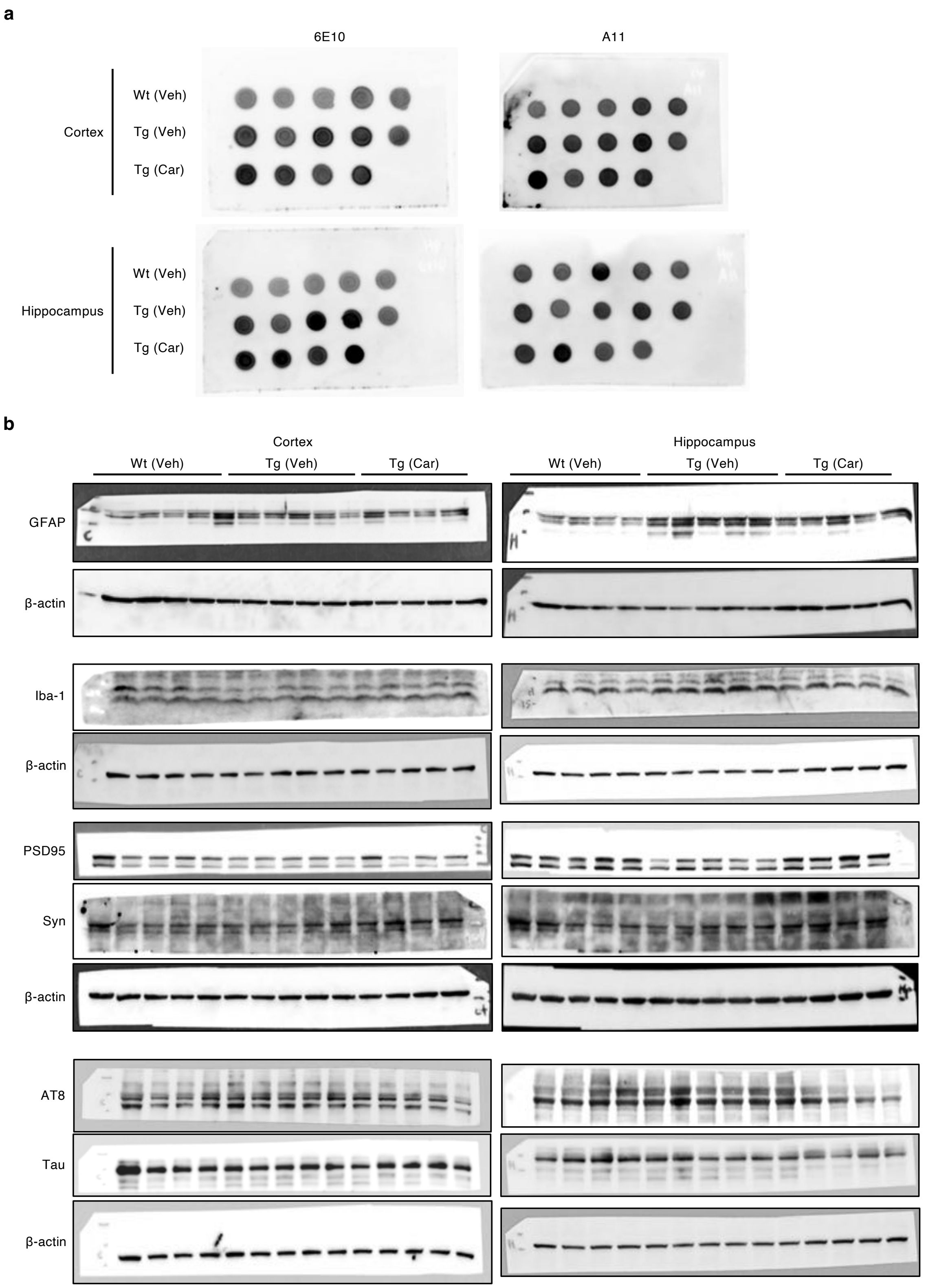

Supplementary Figure S3. Original dot blot and full-membrane images of the immunoblot data with their respective β-actins. (**a**) Dot blot of the cortical and hippocampal brain lysates. (**b**) Western blot analyses assessing the expression levels of Alzheimer-like

characteristics observed in the same membrane. GFAP and Iba-1 were each blotted on separate membranes. PSD95 and synaptophysin were blotted on the same membrane, whereas AT8 and tau were blotted on the same membrane. Wt: Wild-type; Tg: Transgenic; Car: Carprofen; Wt: Wild-type; Veh: Vehicle; Car: Carprofen; GFAP: Glial fibrillary acidic protein; Iba-1: Ionized calcium-binding adaptor molecule 1; PSD95: Post-synaptic density protein 95; Syn: Synaptophysin; AT8: Phosphorylated tau (S202, T205).

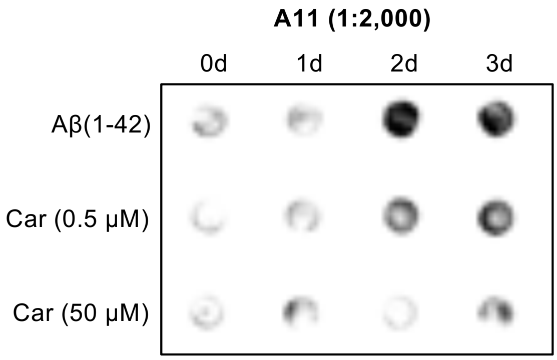

Supplementary Figure S4. Dot blot displaying the intensity of oligomer levels of samples mixed with carprofen (0.5 and 50 μM) and Aβ(1–42) monomer incubated at four different time-points (0-day, 1-day, 2-day, and 3-day) to select the optimal incubation time period for Y-maze test. Non-treated Aβ(1–42) sample was assessed as a control. The oligomeric levels were measured by anti-oligomer antibody A11. Car: carprofen.

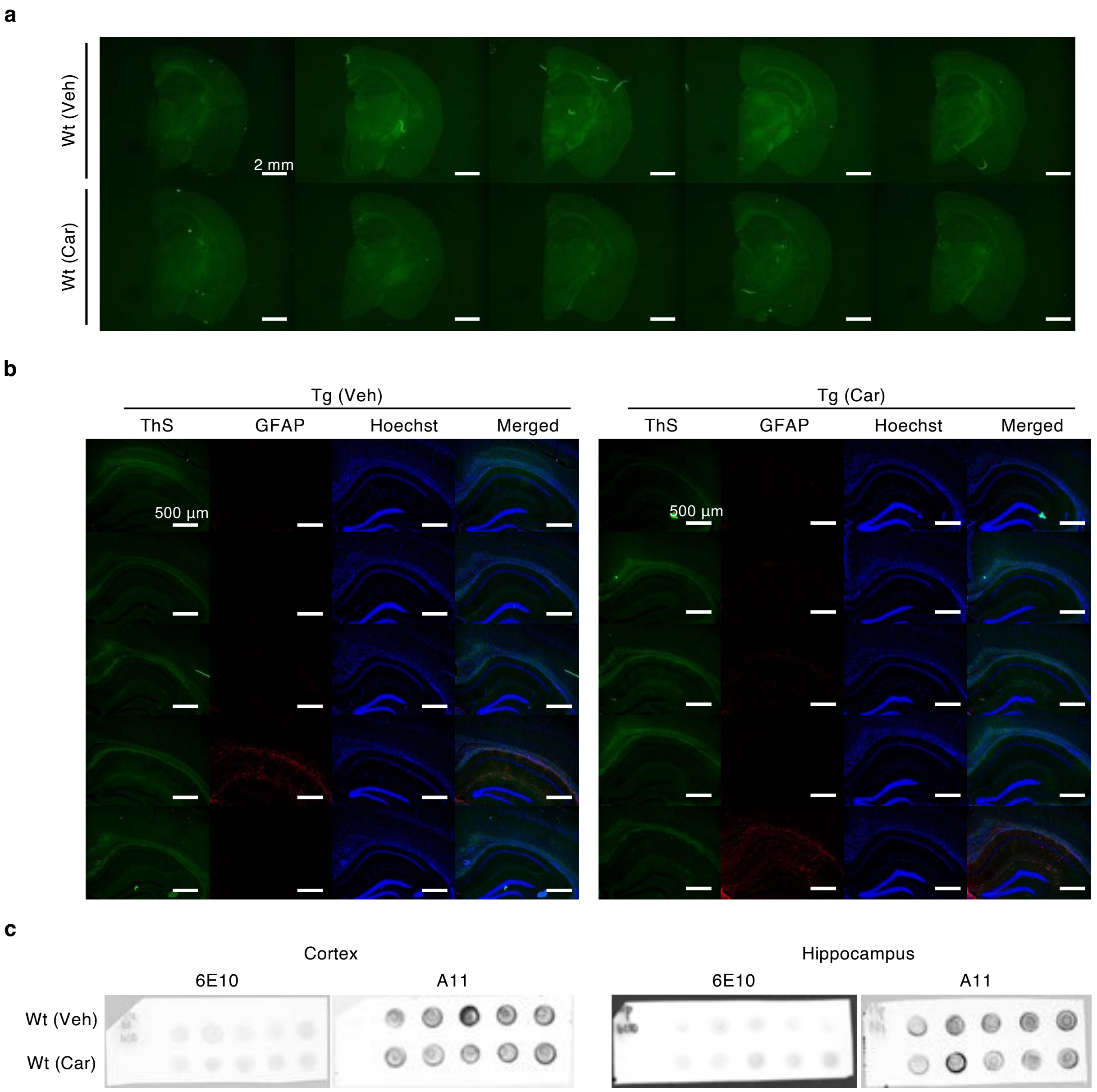

Supplementary Figure S5. Brain images and dot blot of wild-type littermates.

(**a**) Hemisphere images of wild-type littermates treated with vehicle (n = 5) or carprofen (n = 5). Scale bar = 2 mm. (**b**) Hippocampus images of wild-type littermates treated with vehicle (n = 5) or carprofen (*n* = 5). Scale bar = 500 µm. (**c**) Original dot blot and the densitometry of the cortex and hippocampus of wild-type groups treated with vehicle (5% DMSO, n = 5) or carprofen (25 mg/kg/day, n = 5). Wt: Wild-type; Veh: Vehicle; Car: Carprofen; ns: Not significant.

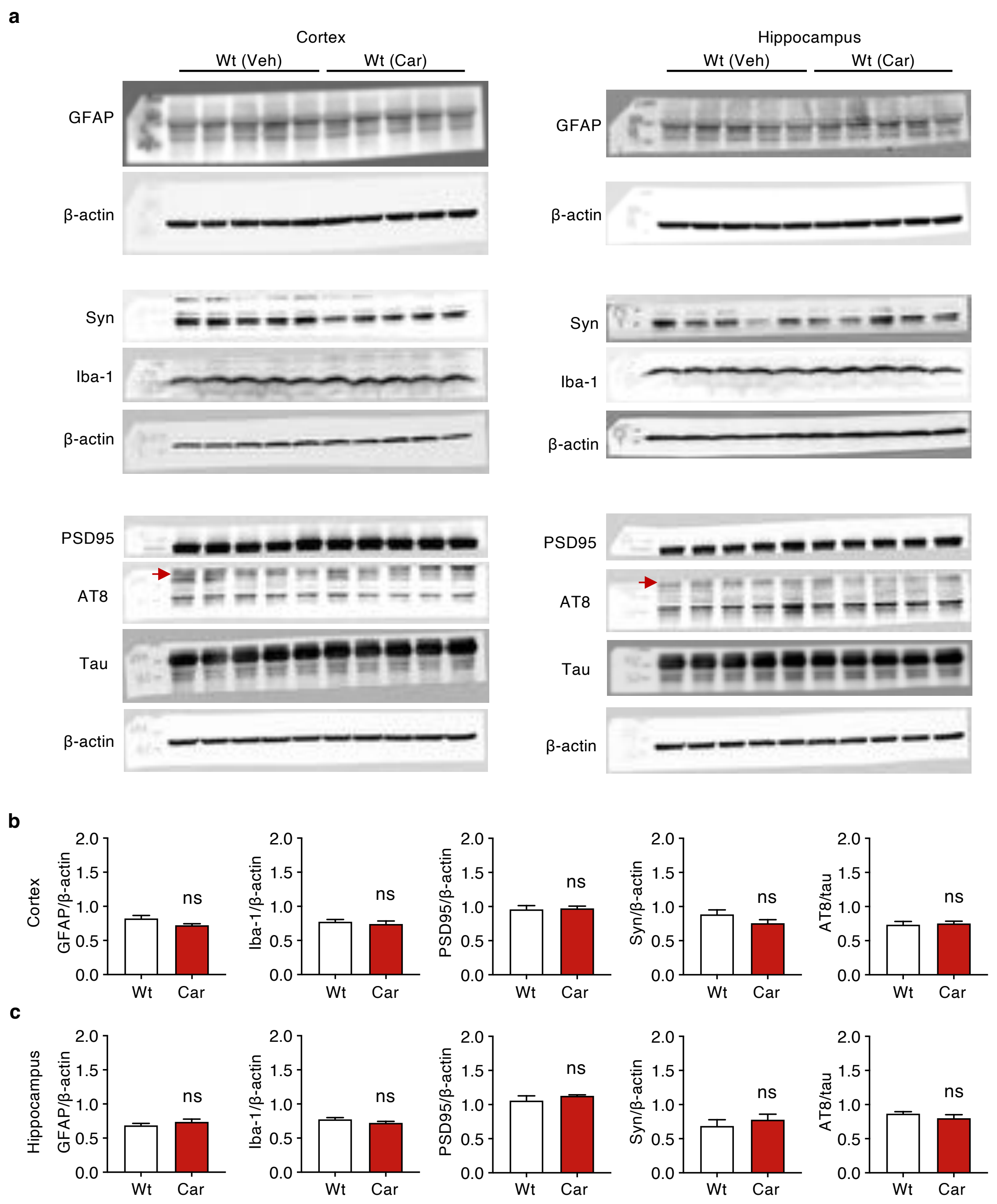

Supplementary Figure S6. Western blot and densitometry of wild-type littermates.

(**a**) Western blot analyses of the cortex and hippocampus of wild-type littermates. (**b,c**) Densitometry measurement of the (**b**) cortex and (**c**) hippocampus of wild-type groups treated with vehicle (5% DMSO, n = 5) or carprofen (25 mg/kg/day, n = 5). All data represent the mean ±SEM and the statistical analyses were performed by two-tailed unpaired *t*-test (densitometry) followed by Bonferroni's post-hoc comparisons tests with the comparison to

the vehicle-treated wild-type littermate group (Wt, white) (*$P < 0.05$, **$P < 0.01$, ***$P < 0.001$, other comparisons not significant). GFAP was blotted on a separate membrane. Synaptophysin and Iba-1 were blotted on the same membrane, whereas PSD95, AT8, and tau were blotted on the same membrane. Wt: Wild-type; Veh: Vehicle; Car: Carprofen; GFAP: Glial fibrillary acidic protein; Iba-1: Ionized calcium-binding adaptor molecule 1; PSD95: Post-synaptic density protein 95; Syn: Synaptophysin; AT8: Phosphorylated tau (S202, T205); ns: Not significant.
